# Genetic analysis of male reproductive success in relation to density in the zebrafish, *Danio rerio*

**DOI:** 10.1186/1742-9994-3-5

**Published:** 2006-04-05

**Authors:** Rowena Spence, William C Jordan, Carl Smith

**Affiliations:** 1 Department of Biology, University of Leicester, University Road, Leicester, UK; 2 Institute of Zoology, Zoological Society of London, Regent's Park, London, UK

## Abstract

**Background:**

We used behavioural and genetic data to investigate the effects of density on male reproductive success in the zebrafish, *Danio rerio*. Based on previous measurements of aggression and courtship behaviour by territorial males, we predicted that they would sire more offspring than non-territorial males.

**Results:**

Microsatellite analysis of paternity showed that at low densities territorial males had higher reproductive success than non-territorial males. However, at high density territorial males were no more successful than non-territorials and the sex difference in the opportunity for sexual selection, based on the parameter *I*_mates_, was low.

**Conclusion:**

Male zebrafish exhibit two distinct mating tactics; territoriality and active pursuit of females. Male reproductive success is density dependent and the opportunity for sexual selection appears to be weak in this species.

## Background

The advent of genetic parentage analysis has had a substantial impact on our understanding of animal mating systems. Many socially monogamous species have proven to be genetically polygamous [1], while territorial or harem-holding males have frequently been shown to be cuckolded [2]. Moreover, due to the operation of sperm competition [3] and cryptic female choice [4], mating success is not equivalent to reproductive success. It is now recognised that genetic analyses are crucial for measuring parentage, and thereby in understanding mating system evolution and the strength of sexual selection [5-7].

The strength of sexual selection depends on the sex difference in the degree of variance in reproductive success for each sex; the greater the difference between the sexes, the more opportunity there is for selection to operate [8]. In most species the variation in male mating success, as defined by mate number and offspring number, exceeds that of female mating success, as defined by clutch size and number of clutches. While females tend to produce similar numbers of offspring, variance in the number of offspring fathered among males can be high, with successful males monopolising females and some males failing to reproduce. The sex difference in the opportunity for sexual selection can be quantified using the parameter *I*_mates _[8]; the difference in male and female variance in reproductive success as a function of the squared mean reproductive success for each sex:

*I*_mates _= *I*_males _- *I*_females_

where



and



if *V *= variance in reproductive success and *X*^*2 *^= squared mean reproductive success.

The greater the value of *I*_mates_, the greater the opportunity for sexual selection, which is typically reflected in the degree of sexual dimorphism. However, variance in male success can be eroded through alternative male mating tactics, sperm competition and cryptic female mate choice [9,10].

The intensity of competition for mates is influenced both by the number of competitors and the temporal and spatial distribution of fertilisations [11,8]. When sexually receptive females are spatially clustered, it is easier for a single dominant male to monopolise matings to the exclusion of other males. In addition, if fertilisations are distributed evenly through time, rather than clustered, a small number of males may be able to monopolise matings. Variance in male reproductive success also correlates negatively with male density. As male density increases, rates of cuckoldry increase and the fitness payoff of territoriality relative to alternative mating tactics declines [2,12,13]. In this study we used behavioural and molecular analysis to investigate the effects of density on male mating success in the zebrafish, *Danio rerio*.

The zebrafish is a small (30–40 mm body length) tropical cyprinid fish, native to the floodplains of North Eastern India and Bangladesh [14]. It is an abundant fish in this region, inhabiting ditches and ponds, where it occurs in small shoals of between 2–30 individuals (R. Spence, personal observation). Males and females are similar in size and colouration. Under laboratory conditions zebrafish breed all year; females spawn every 2–3 days, with all mature ova released during a single spawning bout [15]. Spawning is influenced by photoperiod; mating activity commences within a few minutes of exposure to light following darkness and continues for about one hour [16]. Female zebrafish will release eggs directly onto a bare substrate, but when provided with an artificial spawning site, such as a plastic box filled with gravel or marbles, will preferentially use it for oviposition. Some male zebrafish are territorial during mating and a single male will aggressively attempt to control access of rivals to a spawning site [17]. Courtship in zebrafish involves the male swimming quickly in association with the female, often touching her flanks with his snout, circling tightly in front of her while attempting to lead her to a spawning site. Once over the spawning site, the male swims alongside the female, in close contact but slightly behind her, sometimes oscillating his body at high frequency and low amplitude. Both territorial and non-territorial males show the same courtship behaviour but whereas non-territorial males pursue females all around the aquarium, territorial males confine their activities to within a few body lengths of the spawning site and chase other males away when they try to approach.

In a previous study we manipulated density and sex ratio and measured aggressive and courtship behaviour by territorial males under each treatment [17]. We showed that rates of aggression increased as a function of density, although courtship did not [17]. Here we investigate the effects of density on territorial male reproductive success. We predicted that territorial males would have lower reproductive success at higher densities, due to increased competition from rival males. In addition, in the high-density treatment, we measured the sex difference in the opportunity for sexual selection. For zebrafish, which show little sexual dimorphism, we predicted a low estimate of *I*_mates_.

## Results

**Figure 1:**
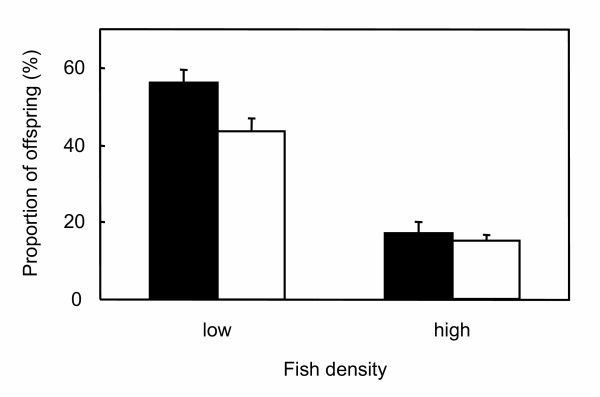
**Territoriality and reproductive success**. The mean proportion (%) + SE of offspring sired by territorial male zebrafish (black bars) and the mean proportion sired per non-territorial male (white bars) under two density treatments.

In each replicate, a single male established a territory around the artificial spawning site and remained in possession of it throughout the 4 days of the experiment. In the low-density treatment territorial males sired a mean ± SD of 56.3% ± 7.58 of the offspring, a significantly greater proportion than non-territorial males at 43.8% ± 7.58 (paired *t*-test: *t*_5 _= 3.05, *P *= 0.028, *d *= 1.76) (Figure 1). In the high-density treatments there was no effect of either territoriality (ANOVA: *F*_1,20 _= 6.39, *P *= 0.304) or sex bias (ANOVA: *F*_1,20 _= 12.3, *P *= 0.159) on the number of offspring sired per male, nor was there an interaction (ANOVA: *F*_1,20 _= 5.21, *P *= 0.352). In the high-density male-biased treatment territorial males sired a mean ± SD of 17.1% ± 12.91 of the offspring, compared to 83.0% ± 12.91 by non-territorial males, a mean of 9.21% ± 9.79 offspring per non-territorial male. In the high-density female-biased treatment the territorial male sired a mean ± SD of 16.9% ± 11.02 of the offspring, compared to 83.3% ± 10.72 by non-territorial males, a mean of 20.8% ± 14.82 per non-territorial male. We then combined both high-density treatments, thereby doubling the sample size; there was still no significant difference in reproductive success between territorial and non-territorial males; territorial males sired a mean ± SD of 17% ± 11.4 of the offspring, compared to 83% ± 11.4 by non-territorial males, a mean of 15% ± 6.39 per non-territorial male (paired *t*-test: *t*_10 _= 0.48, *P *= 0.639, *d *= 0.223) (Figure 1).

**Figure 2:**
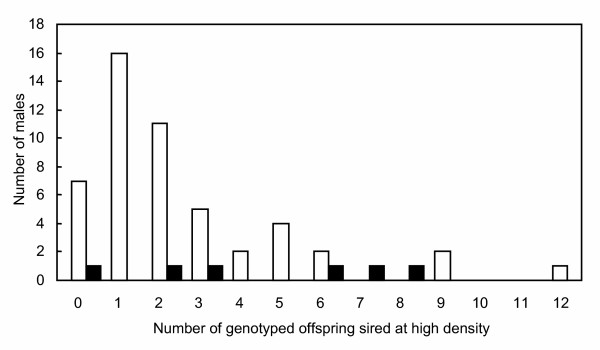
**Distribution of paternity**. The frequency distribution of genotyped offspring among territorial (black bars) and non-territorial (white bars) males in the high-density male-biased treatment.

In the high-density male biased treatment the majority of experimental fish of each sex were represented in the parentage analysis. In total, 87% of males sired at least one of the 30 genotyped offspring (range 0–12, fig. 2). Females produced a mean ± SD of 14.8% ± 3.57 of the offspring each and 89% of the females in each replicate produced at least one of the 30 genotyped offspring. The estimated opportunity for sexual selection was low; *I*_males _= 0.83 ± 0.22 and *I*_females _= 0.80 ± 0.25, giving an estimate of 0.03 for *I*_mates_. In the low density treatment where there was only one female, *I*_females _= 0, so *I*_males _= *I*_mates _= 0.07 ± 0.01.

## Discussion

Despite the wealth of genetic studies conducted on zebrafish, this is the first time a genetic parentage analysis has been applied to understanding their mating system. This study also serves as an example of how genetic analyses can provide insights that are not obvious from behavioural studies alone. We showed that at a low density territorial males sired significantly more offspring than non-territorial males (Fig. 1). While this result is consistent with our previous observation that at low densities territorial males monopolise spawning sites [17], non-territorial males were still able to achieve a relatively high reproductive success. The overall rate of paternity assignment was high (92%) and while it is possible that the paternity of the remaining unassigned 8% could erode the significance of this result, it is unlikely that the pattern of paternity would differ significantly from that observed in the majority that were assigned, given that non-assignment of offspring is likely to be random across potential sires.

At higher densities we detected no significant difference in the reproductive success of territorial and non-territorial males in either sex ratio treatment. We also analysed the combined data from both high-density treatments, thereby doubling the number of replicates, which increased the statistical power of the test and also yielded a non-significant result. While we had expected aggressive territoriality to confer some fitness advantage at higher densities, we had predicted that the advantage would be negatively correlated with density. As the fitness advantage at low densities is not great it is unsurprising that it should be eroded at higher densities. However, this result does raise the question of how territorial behaviour is maintained in the population. Both density levels used here are within the range of densities at which zebrafish occur in nature and territorial defence also occurs under natural conditions (R. Spence & C. Smith, unpublished data). Given that territoriality only occurs during the brief daily spawning period, the possible fitness cost associated with energy expenditure on territoriality may not exceed that for courtship. Consequently, while territorial defence confers a fitness advantage at low densities, it may not always do so at high densities, though the reproductive fitness of territorial males may be at least equal to that of non-territorial males. In addition, the adoption of one or other tactic, territorial defence or active pursuit of females, may be frequency dependent, in which case they would be predicted to confer equal fitness payoffs.

Alternative mating tactics are common in fish, partly because of the prevalence of external fertilisation, which makes it hard to exclude rivals [18]. In genetic studies of several nest-tending species with paternal care Dewoody & Avise [2] found that between 5–30% of embryos in nests were not sired by the nest owner. In some species, such as Atlantic salmon, *Salmo salar*, and bluegill sunfish, *Lepomis macrochirus*, territorial and sneaker males are morphologically distinct [19]. In other species, such as the three-spined stickleback *Gasterosteus aculeatus *[20] and the European bitterling, *Rhodeus sericeus *[13,21] the adoption of one or other role is flexible and probably frequency dependent. Although each territorial male in this study maintained his territorial role throughout the 4 days of the experiment, territorial male zebrafish are not morphologically distinct. Further, on the basis of laboratory and field observations, the frequency of territorial behaviour is influenced by the availability of high quality spawning sites, a factor that was not manipulated in the current study (R. Spence & C. Smith, unpublished data).

It should be noted that fertilisation rates in zebrafish may not always reach 100%, though this was not recorded in the current study. Thus, our results reflect the reproductive success of males as determined by differential embryo survival to hatching (three days post-fertilisation) rather than fertilisation success. Our genetic analysis also showed that the majority of females in each replicate produced offspring. We previously showed that females produce fewer eggs *per capita *at high densities [17] but in the absence of paternity analysis it was not clear whether this was because each female produced fewer eggs, or whether some females were excluded from spawning. This analysis suggests that females spawn smaller clutches at higher densities.

Our estimate of *I*_mates _indicates that there is a low opportunity for sexual selection in zebrafish; there was no significant difference in the variance in reproductive success between males and females. Because our estimate is based on genetic parentage data, it takes account of both behavioural differences (such as territoriality) and post-mating forms of sexual selection (sperm competition and cryptic female choice) which can impact on variance in reproductive success. This result is consistent with the lack of a marked sexual dimorphism in this species. Variance in male mating success is dependent on the temporal and spatial clustering of females; the opportunity for selection is predicted to be highest when receptive females show low temporal but high spatial clustering [8]. Although zebrafish spawn almost daily under laboratory conditions, spawning is confined to an approximately 1-hour period each day; i.e. matings are highly temporally clustered. In nature, spawning is more seasonal, but is similarly largely confined to a brief period at dawn (R Spence & C Smith, personal observation). Spatial clustering of females may occur where sites for oviposition are limiting. However, although both females and males discriminate among spawning locations (Spence & Smith, unpublished data) they use a broad range of oviposition sites. Consequently, under natural conditions males may not always be able to monopolise either receptive females or sites of reproduction, with the outcome that the opportunity for sexual selection is weak.

## Conclusion

The results of this study suggest that zebrafish have a promiscuous mating system with a low opportunity for sexual selection. Parentage analysis indicates that territoriality confers a fitness benefit at low densities, but at higher densities has an equivalent fitness payoff to non-territorial behaviour.

## Methods

### Experiment

We carried out a behavioural experiment in February 2004, using 216 zebrafish obtained from a commercial supplier. The experimental design and results of behavioural analyses are presented in [17], though no results are repeated here. The original experimental design comprised two factors: density and sex ratio. However, only density effects were investigated in the present study. The low-density treatment consisted of three fish, one female and two males (six replicates) and the high-density treatment of fifteen fish, either ten males and five females or five males and ten females (six replicates of each).

Fish were housed in an environmentally controlled room with a 14:10 h hour light: dark cycle. Experiments were conducted in 60 l glass aquaria (60 × 35 cm and 40 cm high) with rested tap water at 26.5 – 28.5°C. Water quality in aquaria was maintained using filters and aquaria water was aerated with an air stone. Males and females were randomly assigned to experimental treatments, females being distinguished by the presence of a genital papilla. We placed opaque dividers between aquaria to prevent visual interactions between neighbouring fish. Fish were fed three times each day with a mixture of frozen bloodworm and commercial salmon smolt pellets. A single plastic box (150 × 100 mm and 40 mm deep) filled with 150 small glass marbles, was placed in the front right hand corner of every experimental aquarium as a spawning site. Zebrafish readily use boxes of marbles for oviposition; the eggs fall among the marbles, which prevent egg cannibalism.

Each replicate lasted for four days. We observed each aquarium daily during the spawning period to determine whether there was a single, dominant male defending the spawning site, and whether the same male remained territorial throughout. Individual territorial males were identified from unique features of their colour pattern. A subset of the replicates was videotaped for 5 min each morning and the frequency of aggressive and courtship behaviours by the territorial male were scored from video footage. Spawning sites were removed daily after the end of the spawning period and all the eggs deposited in them were carefully removed, counted and incubated until hatching, whereupon the embryos were preserved in 95% ethanol. Spawning sites were replaced 3–4 h after removal.

On the fifth day following the start of the experiment, all experimental fish were removed from aquaria and killed with an overdose of anaesthetic. Fish were measured to the nearest 0.1 mm, dissected to confirm sex and fin clips taken for parentage analysis.

### Parentage analysis

We genotyped all the adults and a subset of 31–32 offspring from each replicate. The mean (± SD) daily number of eggs per replicate was 52 ± 8.84 in the low density treatment and 270 ± 28.5 in the high density treatments. A total of 198 adults and 560 offspring were genotyped. Offspring from a single day's spawning were selected haphazardly from each replicate. DNA was extracted from fin samples or embryos with the yolk sac removed, using Promega Wizard SV 96 Genomic DNA purification system.

Individuals were screened across seven microsatellite loci (Z10914, Z1213, Z1233, Z1496, Z6454, Z669 and Z851) belonging to six different linkage groups, with a mean of 7 alleles per locus. Primer sequences were taken from the zebrafish genetic map website [22-24]. DNA was amplified in a 6 μl reaction volume containing 1 μl genomic DNA; 2 pmol dye-labelled forward primer; 2 pmol reverse primer and 4 μl Qiagen Multiplex PCR Master Mix. PCR cycling consisted of an initial 12 min denaturation at 95°C, 30 cycles of 30 s at 94°C, 2 min at 58°C and 1 min at 72°C and a final extension at 60°C for 30 s. 1 μl PCR product was mixed with 10 μl dionized formamide and 15 μl LIZ 500 size standard and run on an automated sequencer, Applied Biosystems 3100 Genetic Analyzer. Fragment length was determined using Genemapper 3.5 software. All adults were genotyped 3 times in order to control for the effects of null alleles and allelic dropout [25]. The error rate (the ratio of observed allelic differences to total allelic comparisons among repeated amplifications) was estimated to be 3.4%.

Parentage was assigned using CERVUS 2.0 software [26]. Observed heterozygosities ranged from 0.20 to 0.71. In the low-density treatment, where maternal genotype was known, paternity was assigned with 95% confidence in 92% of the offspring sampled, (range 78%–100% between replicates). In the high-density treatments where maternity was not known, CERVUS was used in a stepwise manner, determining parentage first for the sex with the smallest number of candidate parents in a given replicate (i.e. females in the male-biased treatment and males in the female-biased treatment) and using those data where parentage was assigned with 95% confidence to determine parentage for the other sex [26]. Paternity was assigned with 95% confidence to 244 of 380 offspring genotyped and with 80% confidence to a further 107, (range 60%–100% between replicates). Maternity was assigned with 95% confidence to 205 of the offspring and with 80% confidence to a further, 137, (range 78%–100% between replicates). The combined exclusionary power for the high-density treatments was thus 83%.

### Data analysis

We tested all data for normality using a Kolmogorov-Smirnov test and for equality of variance using Bartlett's test. The reproductive success of males was calculated as a percentage of offspring sired within a replicate, paternity being assigned for each individual male, both territorial and non-territorial. Only data where paternity could be assigned with at least 80% confidence were used in the analysis. A two factor ANOVA was used to test for the effects of territoriality and sex bias on male reproductive success in the high density treatments. We used paired *t*-tests to test for a difference in reproductive success between territorial and non-territorial males (mean value per male) within each density level. Following the recommendation of Nakagawa [27], Bonferroni corrections were not applied and instead a measure of effect size (Cohen's *d*) was estimated. This index measures the magnitude of a treatment effect as the standardised difference between two means by comparing the overlap in the distribution between the two data sets independently of sample size. An effect size of 0.8 is defined as large [28].

## Authors' contributions

RS and CS designed the study, RS conducted the experiment and parentage analysis, analysed the data and wrote the paper, WCJ oversaw the genetic work.
